# A Mathematical Model of a Piezo-Resistive Eight-Beam Three-Axis Accelerometer with Simulation and Experimental Validation

**DOI:** 10.3390/s18113641

**Published:** 2018-10-26

**Authors:** Jinlong Song, Changde He, Renxin Wang, Chenyang Xue, Wendong Zhang

**Affiliations:** Science and Technology on Electronic Test and Measurement Laboratory, North University of China, Taiyuan 030051, China; b1506015@st.nuc.edu.cn (J.S.); hechangde@nuc.edu.cn (C.H.); xuechenyang@nuc.edu.cn (C.X.)

**Keywords:** mathematical model, piezo-resistive eight-beam three-axis accelerometer, working principle, static and dynamic performance, accurate and rapid way

## Abstract

A mathematical model of a sensor is vital to deeply comprehend its working principle and implement its optimal design. However, mathematical models of piezo-resistive eight-beam three-axis accelerometers have rarely been reported. Furthermore, those works are largely focused on the analysis of sensing acceleration in the normal direction, rather than in three directions. Therefore, a complete mathematical model of a piezo-resistive eight-beam three-axis accelerometer is developed in this paper. The validity of the mathematical model is proved by a Finite Element Method (FEM) simulation. Furthermore, the accelerometer is fabricated and tested. The prime sensitivities of X, Y and Z axes are 0.209 mV/g, 0.212 mV/g and 1.247 mV/g at 160 Hz, respectively, which is in accord with the values obtained by the model. The reason why the prime sensitivity S_ZZ_ is bigger than S_XX_ and S_YY_ is explained. Besides, it is also demonstrated why the cross-sensitivities S_XZ_ and S_YZ_ exceed S_ZX_ and S_ZY_. Compared with the FEM model, the developed model could be helpful in evaluating the performance of three-axis accelerometers in an accurate and rapid way.

## 1. Introduction

Micro-electro-mechanical system (MEMS) accelerometers have been widely applied in many applications, such as military, industrial, medical, and consumer devices [1,2,3,4,5,6]. According to their different principles of sensing acceleration, MEMS accelerometers can be divided into piezo-resistive, piezoelectric, capacitive, etc. [7,8,9]. A comparison of the three types of accelerometers is shown in Table 1. Compared with capacitive and piezoelectric accelerometers, piezo-resistive accelerometers feature quick responses, simple processing and detection circuits, and good direct current (DC) responses. Their shortcoming is the susceptibility to temperature changes. Since they were first developed by Roylance and Angell [10], many scholars have done a lot of work about piezo-resistive accelerometers [11,12,13,14].

Its mathematical model is the foundation to comprehend the working principle of a sensor. It is also helpful for the optimal design of a sensor. Predecessors have done much in this respect, as shown in Table 2. Nevertheless, so far, very little information about the mathematical model of piezo-resistive eight-beam three-axis accelerometers is available, and only when acceleration is in the normal (Z) direction, not in three directions (X, Y and Z). For example, Kampen and Wolffenbuttel gave an analysis of static modeling (sensitivity and cross-axis sensitivity) and dynamic behavior such as resonance and damping of structure that with a proof-mass and four or eight beams. Their work mainly focused on the analysis of the normal direction [15]. In 2015, Mukhiya et al. modeled a 1-Dof MEMS bulk micro-machined piezo-resistive accelerometer similar to the work reported in [15], the difference being that a FEM-based simulation with CoventorWare was conducted to validate the model [16]. Yu et al. developed mathematical model of a structure consisting of two masses, four suspending piezo-resistive beams (SPBs), two supporting beams and one hinge. The effects of various geometrical parameters on the stress and natural frequency were compared in theory and simulation [13]. Lin et al. established an analytical model of a symmetric beam-mass structure based on the mechanics of the materials and vibration theory, however, the model was applicable only when acceleration was in the normal direction [17]. Wan and Yan analyzed the natural frequency, stress, strain and sensitivity of a two-end fixed beam piezo-resistive accelerometer structure. Based on the model, the influence of structure parameters on its natural frequencies was obtained [18]. Meng et al. developed a model to analyze the structure mechanics of a piezo-resistive three-axis accelerometer. However, their model only analyzed the displacement, stress and natural frequency in the case of acceleration in the normal direction [19]. Wang et al. developed a model of a piezo-resistive micro-accelerometer where the proof-mass was suspended by four sensing beams and four suspension beams. The model can predict the displacement, stress and natural frequency, but not sensitivity, cross-axis sensitivity and amplitude frequency characteristics [20]. Hang et al. developed an accelerometer made of a heavy proof-mass and four long beams to obtain high resolutions by reducing the resonance frequency. Electronics sensitivity and frequency responses were modeled [21]. Liu et al. presented a theoretical model of a high-g accelerometer as a crossed clamped-clamped Timoshenko beam with a lumped moment of inertia at the free end. 

For the reason that an accelerometer with a proof-mass supported by eight beams has the following advantages: (1) full bridge differential amplification leading to high sensitivity; (2) high natural frequency; (3) acceleration measurement in three axes, the study of this structure is of great significance. Nevertheless, almost all previous modeling work were concerned only with acceleration in the normal (Z) direction, hence, it is necessary to supplement the research. As a result, a mathematical model of piezo-resistive eight-beam three-axis accelerometer is developed. The performance of prime sensitivities, cross-axis sensitivities and natural frequency are analyzed based on the model. The model is validated by FEM and testing results of a designed and fabricated accelerometer.

## 2. Modeling Piezo-Resistive Eight-Beam Three-Axis Accelerometer

**Figure 1:**
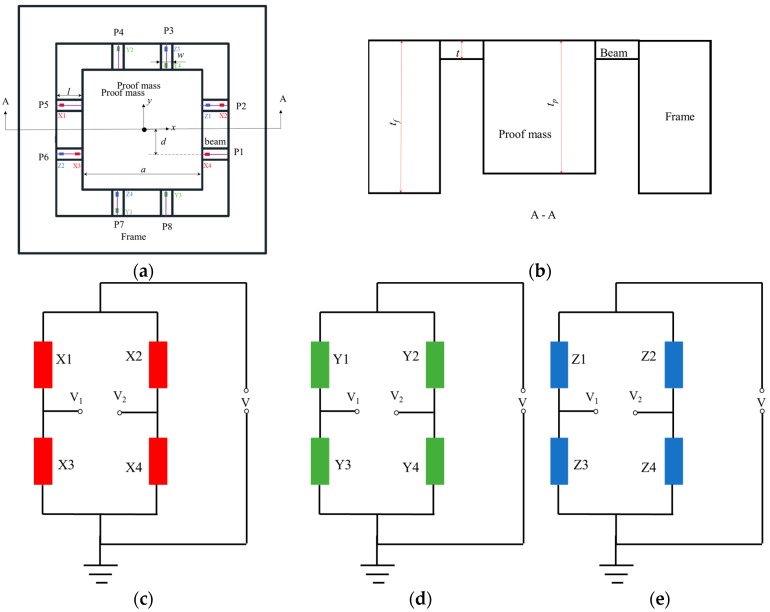
(**a**) Top view of accelerometer (**b**) cross section of accelerometer (**c**) X Wheatstone bridge (**d**) Y Wheatstone bridge (**e**) Z Wheatstone bridge.

The structure of a piezo-resistive eight-beam three-axis accelerometer consisting of a proof-mass, eight beams and a frame is illustrated in Figure 1a,b. The upper surfaces of the beam, proof-mass and frame are in the same plane. There are two parallel rectangular beam on each side of the proof-mass. The proof-mass is suspended on the frame through beams. P type piezo-resistors are fabricated on the surface of beam end by doping boron. Four ingeniously placed piezo-resistors marked with red, green and blue form a Wheatstone bridge for detecting acceleration in the X, Y and Z direction, respectively, as shown in Figure 1c–e.

In a Wheatstone bridge, the resistance changes are directly converted to a voltage signal. Considering the feature of this structure, the model can be divided into two circumstances: acceleration in the Z and X (or Y) direction. For the convenience of later analysis, beams along the X and Y axis are defined as X-beams and Y-beams, respectively. The Wheatstone bridge for detecting acceleration in X, Y and Z direction is called the X, Y and Z Wheatstone bridge, respectively.

### 2.1. Modeling the Static Behavior

#### 2.1.1. Acceleration in Normal (Z) Direction

Under an external acceleration $a_{z}$ in the normal direction, the proof-mass m would move down or up. Due to the structure symmetry, the eight beams share the same deformation. The bending moment *M*_1_(*x*) and displacement $w_{1}\left(x\right)$ of the beam at location *x* (from frame to proof-mass) can be expressed as:

(1) $$M_{1}\left(x\right)=M_{1}-F_{R1}\left(l-x\right)$$

(2) $$w_{1}\left(x\right)=\iint_{0}^{x}\left(\frac{M_{1}\left(x\right)}{EI}dx\right)dx+Cx+D$$

where *M*_1_ is the reaction moment; *F_R_*_1_ is the reaction force, $F_{R1}=ma_{z}/8;$ l is the beam length; *E* is Young’s modulus; *I* is the moment of inertia, $I=wt^{2}/12$; *w* is beam width; *t* is beam thickness; *C* and *D* are constant.

The boundary condition for these equations are:

(3) $$w_{1}\left(0\right)=w_{1}^{\prime }\left(0\right)=0$$

From Equations (1)–(3), we can get the expressions of generated displacement and stress on the surface of beam along longitudinal direction:

(4) $$w_{1}\left(x\right)=\frac{2x-3l}{96EI}x^{2}ma_{z}$$

(5) $$\sigma_{1l}=\frac{3\left(l/2-x\right)}{4wt^{2}}ma_{z}$$

#### 2.1.2. Acceleration in X (or Y) Direction

**Figure 2:**
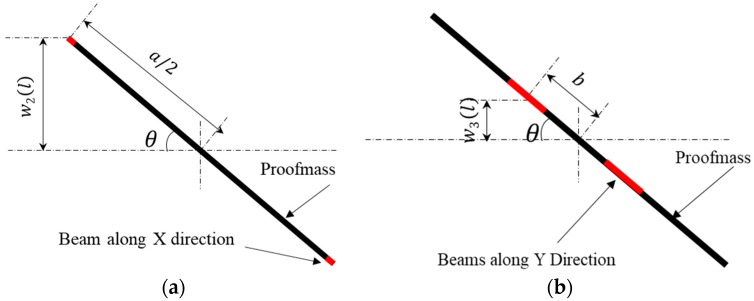
Movement of proof-mass when acceleration in X direction (**a**) for analysis of beams along X direction (**b**) for analysis of beam along Y direction.

Due to the symmetry of the accelerometer structure, static behavior results under an external acceleration in X direction are equivalent to the sensor being rotated by 90 degrees counterclockwise under an external acceleration in the Y direction. Consequently, results under an external acceleration in the X direction are analyzed in the following sections. For this situation, the movement of the proof-mass is illustrated in Figure 2a,b. For the reason that the thickness of the beam is only a few microns or tens of microns, the width of the beam is generally hundreds of microns. The section modulus in bending of Y-beams in plane is dozens of times that of the out of plane bending, therefore, to simplify the analysis, the in plane bending of Y-beams is neglected. The whole inertia force of proof-mass acts on the X-beams. 

##### Beams Along the X Direction

X-beams can be divided into two kinds: the left two X-beams and the right two X-beams. They are subjected to both bending moment and tension/compression. It is obvious that the displacement and stress of left two X-beams and the right two X-beams share the same value but opposite sign. Therefore, just the left two X-beams are analyzed. The bending moment *M*_2_(*x*), displacement $w_{2}\left(x\right)$ at location *x* (from fixed end to proof-mass end) can be expressed as:

(6) $$M_{2}\left(x\right)=-M_{2}+F_{R2}\left(l-x\right)$$

(7) $$w_{2}\left(x\right)=\iint_{0}^{x}\left(\frac{M_{2}\left(x\right)}{EI}dx\right)dx+Cx+D$$

where *M*_2_ is reaction moment; *F_R_*_2_ is reaction force.

The boundary condition for these equations are:

(8a) $$w_{2}\left(0\right)=w_{2}^{\prime }\left(0\right)=0$$

(8b) $$\left|w_{2}\left(l\right)\right|=\left|bw_{2}^{\prime }\left(l\right)\right|$$

where *b* is half of the length of the proof-mass.

From Equations (6)–(8), the following expressions can be obtained:

(9) $$F_{R2}=\frac{3\left(2b+l\right)}{l\left(2l+3b\right)}M_{2}$$

(10) $$w_{2}\left(x\right)=\frac{1}{EI}(-\frac{1}{2}M_{2}x^{2}+\frac{1}{2}F_{R2}lx^{2}-\frac{1}{6}F_{R2}x^{3})$$

##### Beams Along the Y Direction

Compared to the X-beams, Y-beams are subject to bending and torsion. Like the X-beams, Y-beams can also share the same value but opposite sign of displacement and stress. As a result, only the left two Y-beams are studied in this section. The bending moment *M*_3_(*x*) and displacement $w_{3}\left(x\right)$ at location *x* (from fixed end to proof-mass end) can be expressed as:

(11) $$M_{3}\left(x\right)=-M_{3}+F_{R3}\left(l-x\right)$$

(12) $$w_{3}\left(x\right)=\iint_{0}^{x}\left(\frac{M_{3}\left(x\right)}{EI}dx\right)dx+Cx+D$$

where *M*_3_ is the reaction moment; *F_R_*_3_ is the reaction force.

The boundary condition for these equations are:

(13a) $$w_{3}^{\prime }\left(0\right)=w_{3}^{\prime }\left(l\right)=0$$

(13b) $$w_{3}\left(0\right)=0$$

(13c) $$\left|w_{3}\left(l\right)|=|dw_{2}^{\prime }\left(l\right)\right|$$

where *d* is half of the distance between the two parallel beams. *d* is half of the length of the proof-mass. From Equations (11)–(13), the following expressions can be obtained:

(14) $$M_{3}=\frac{1}{2}F_{R3}l$$

(15) $$M_{3}=\frac{3d}{2l+3b}M_{2}$$

The twist angle of beams along Y direction at location *x* = *l* can be expressed as Equation (16) when w/t>10:

(16) $$\varphi =\frac{6Tl\left(1+\mu \right)}{Ewt^{3}}$$

where *T* is the torsion, μ is the Poisson ratio.

Applying boundary conditions that the proof-mass $\left|w_{2}^{\prime }\left(l\right)\right|=\left|\varphi \right|$, the following equation can be obtained:

(17) $$T=\frac{\varepsilon l}{2l+3b}M_{2}$$

where $\varepsilon =\frac{1}{1+\mu }.$

##### Proof-Mass

When the proof-mass is taken as a research object, the moment balance equation can be expressed as

(18) $$4\left(F_{R2}b+M_{2}\right)+4\left(F_{R3}b+T\right)=M$$

where $M=mha_{X}/2$; *h* is the thickness of the proof-mass.

Substituting Equations (9), (14), (15) and (17) into Equation (18), the reaction moment *M*_2_ can be expressed by:

(19) $$M_{2}=\frac{l\left(2l+3b\right)}{4\left[6b^{2}+6d^{2}+\left(2+\varepsilon \right)l^{2}+6bl\right)]}M=\frac{\left(2l+3b\right)lh}{8\left[6b^{2}+6d^{2}+\left(2+\varepsilon \right)l^{2}+6bl\right]}ma_{X}$$

Substituting Equations (9) and (19) into Equation (6), the reaction moment $M_{2}\left(x\right)$ can be expressed as:

(20) $$M_{2}\left(x\right)=\frac{h\left[l^{2}+3bl-3x\left(2b+l\right)\right]}{8\left[6b^{2}+6d^{2}+\left(2+\varepsilon \right)l^{2}+6bl\right)]}ma_{X}$$

Substituting Equations (14), (15) and (19) into Equation (11), the reaction moment $M_{3}\left(x\right)$ can be expressed as:

(21) $$M_{3}\left(x\right)=\frac{3dh\left(l-2x\right)}{4\left[6b^{2}+6d^{2}+\left(2+\varepsilon \right)l^{2}+6bl\right)]}ma_{X}$$

The stress at location *x* on the surface of left two X-beams can be expressed as:

(22) $$\sigma_{2l}=\frac{M_{2}\left(x\right)}{W}=-\left[\frac{3h\left[l^{2}+3bl-3x\left(2b+l\right)\right]}{4wt^{2}\left[6b^{2}+6d^{2}+\left(2+\varepsilon \right)l^{2}+6bl\right)]}+\frac{1}{4wt}\right]ma_{X}$$

where *W* is section modulus in bending, $W=wt^{2}/6$.

The stress at location *x* on the surface of left two Y-beams is given by:

(23) $$\sigma_{3l}=\frac{M_{3}\left(x\right)}{W}=\frac{9dlh\left(2x-l\right)}{4lwt^{2}\left[6b^{2}+6d^{2}+\left(2+\varepsilon \right)l^{2}+6bl\right)]}ma_{X}$$

### 2.2. Output and Sensitivity

For higher piezo-resistive coefficients in the <110> directions of silicon (100), boron doped (p-type) resistors are preferred over phosphorous doped (n-type) resistors [22]. From simulation, the ratio of two normal stresses to longitudinal stress becomes bigger. This phenomenon will decrease the sensitivity of the sensor. To eliminate this effect, the position of piezo-resistors should have a certain distance from the ends. Therefore, the relative change of piezo-resistors can be expressed as [15,21]:

(24) $$\frac{\Delta R}{R}=\pi_{l}\sigma_{l}$$

It is assumed that all piezo-resistors are of same value (*R*) and placed symmetrically. For acceleration in the X direction, the induced stress experienced by resistors and relative changes in resistance are summarized in Table 3. The symbol ‘+’ indicates the increase of resistance while ‘−’ represents decrease. Output voltages $V_{MN}$ (first subscripts *M* denotes the Wheatstone bridge and second subscripts *N* stands for direction of an external acceleration) for acceleration in X direction are given by Equations (25). For acceleration in the Z direction, induced stress experienced by resistors and changes in resistance are summarized in Table 4. Output voltages $V_{MN}$ for acceleration in X direction are given by Equation (26):

(25a) $$V_{XX}=\left(\frac{1+\Delta R_{X3}/X3}{2-\Delta R_{X1}/X1+\Delta R_{X3}/X3}-\frac{1-\Delta R_{X4}/X4}{2+\Delta R_{X2}/X2-\Delta R_{X4}/X4}\right)V$$

(25b) $$V_{YX}=\left(\frac{1-\Delta R_{3}/Y3}{2-\Delta R_{1}/Y1-\Delta R_{3}/Y3}-\frac{1-\Delta R_{4}/Y4}{2-\Delta R_{2}/Y2-\Delta R_{4}/Y4}\right)V$$

(25c) $$V_{ZX}=\left(\frac{1+\Delta R_{3}/Z3}{2-\Delta R_{1}/Z1+\Delta R_{3}/Z3}-\frac{1+\Delta R_{4}/Z4}{2-\Delta R_{2}/Z2+\Delta R_{4}/Z4}\right)V$$

(26a) $$V_{XZ}=\left(\frac{1-\Delta R_{X3}/X3}{2+\Delta R_{X1}/X1-\Delta R_{X3}/X3}-\frac{1-\Delta R_{X4}/X4}{2+\Delta R_{X2}/X2-\Delta R_{X4}/X4}\right)V$$

(26b) $$V_{YZ}=\left(\frac{1-\Delta R_{3}/Y3}{2+\Delta R_{1}/Y1-\Delta R_{3}/Y3}-\frac{1-\Delta R_{4}/Y4}{2+\Delta R_{2}/Y2-\Delta R_{4}/Y4}\right)V$$

(26c) $$V_{ZZ}=\left(\frac{1+\Delta R_{3}/Z3}{2-\Delta R_{1}/Z1+\Delta R_{3}/Z3}-\frac{1-\Delta R_{4}/Z4}{2+\Delta R_{2}/Z2-\Delta R_{4}/Z4}\right)V$$

The sensitivity is defined by the ratio of output voltage over input acceleration. The sensitivity of a three axis accelerometer includes the prime axis sensitivity and cross-axis sensitivity. $S_{XX}$, $S_{YY}$ and $S_{ZZ}$ mean prime axis sensitivity, while $S_{YX}$, $S_{ZX}$, $S_{XY}$, $S_{ZY}$, $S_{XZ}$ and $S_{YZ}$ indicate the cross-axis sensitivity.

### 2.3. Modeling Dynamic Behavior

#### 2.3.1. Natural Frequency

The displacement of a proof-mass *z* is equal to that of beam at location *x* = *l* when the external acceleration in normal direction:

(27) $$z=w_{1}\left(l\right)=-\frac{l^{3}}{8Ewt^{3}}ma_{Z}$$

According to Newton’s second law $ma_{Z}-k_{z}z=0$, the spring constant $k_{z}$ can be expressed as:

(28) $$k_{z}=\frac{8Ewt^{3}}{l^{3}}$$

As a result, the first order natural frequency is given by:

(29) $$f_{1}=\frac{1}{2\pi }\sqrt{\frac{k_{z}}{m}}=\frac{1}{\pi }\sqrt{\frac{2Ewt^{3}}{ml^{3}}}$$

The rotation angle of proof-mass equals to rotation angle of beams at location *x* = *l* when the external acceleration in X (or Y) direction:

(30) $$\theta =w_{2}^{\prime }\left(l\right)=\frac{1}{EI}\left(-M_{2}L+\frac{1}{2}F_{R2}l^{2}\right)$$

Substituting Equations (9) and (19) in Equation (30), the following expression can be obtained:

(31) $$M=-\frac{8Ewt^{3}\left[6b^{2}+6d^{2}+\left(2+\varepsilon \right)l^{2}+6bl\right]}{l^{3}}w_{2}^{\prime }\left(l\right)$$

So, the spring constant $k_{X}$ for this condition is:

(32) $$k_{X}=\frac{2Ewt^{3}\left[6b^{2}+6d^{2}+\left(2+\varepsilon \right)l^{2}+6bl\right]}{3L^{3}}$$

Hence, the secondary and third order frequency can be expressed as:

(33) $$f_{2,3}=\frac{1}{2\pi }\sqrt{\frac{k_{X}}{m_{eq}}=}\frac{1}{2\pi }\sqrt{\frac{8Ewt^{3}\left[6b^{2}+6d^{2}+\left(2+\varepsilon \right)l^{2}+6bl\right]}{4\rho l^{3}a^{2}\left(h^{3}-3h^{2}\delta +3h\delta^{2}\right)+\rho hl^{3}a^{4}}}$$

where $m_{eq}=\frac{1}{3}\rho a^{2}[h^{3}-3h^{2}\delta +3h\delta^{2}]+\frac{1}{12}\rho a^{4}h$; δ=t/2.

#### 2.3.2. Amplitude Frequency Characteristics

Damping significantly affects the dynamic performance of the vibration system. For the reason that a decrease of mechanical size will lead to an increase of damping ratio, damping of this MEMS accelerometer can’t be neglected. There are two main sources of damping for MEMS accelerometers, one is the structural damping of each structure and the other is air damping. The damping force introduced by the structure is so small compared with the air damping that it could be neglected. Squeeze-film damping is the main air damping and its damping coefficient c can be expressed as [23,24]:

(34) $$c=\beta \left(\frac{B}{L}\right)\frac{\mu LB^{3}}{h_{a}^{3}}$$

where *μ* is viscosity factor of air, *μ* = 1.82 × 10^−5^ kg/m·s; *L* is the length of proof-mass; *B* is the width of proof-mass; *h_a_* is the thickness of air damping; $\beta \left(\frac{W}{L}\right)$ is correction factor determined by the width and length of rectangular plates and is given by:

(35) $$\beta \left(\frac{W}{L}\right)\approx 1-0.76\left(\frac{W}{L}\right)+0.16{\left(\frac{W}{L}\right)}^{2}$$

The damping ratio ξ of accelerometer is given by:

(36) $$\xi =\frac{c}{2\sqrt{km}}$$

The amplitude characteristics of this secondary order system can be expressed as [25]:

(37) $$\frac{H\left(j\omega \right)}{K}=\frac{1}{\sqrt{{\left[1-{\left(\frac{\omega }{\omega_{0}}\right)}^{2}\right]}^{2}+4\xi^{2}{\left(\frac{\omega }{\omega_{0}}\right)}^{2}}}$$

where ω is the angular frequency of the input acceleration; $\omega_{0}$ is the natural angular frequency of the undamped system; *K* is the static sensitivity.

## 3. Mechanical Simulation

To validate the mathematical model developed in this paper, finite element method (FEM) simulations are conducted. The structure parameters used for simulation are listed in Table 5. Based on the above analysis, only the external acceleration in the X direction is simulated.

### 3.1. Static Simulation

#### 3.1.1. Acceleration in Normal (Z) Direction

**Figure 3:**
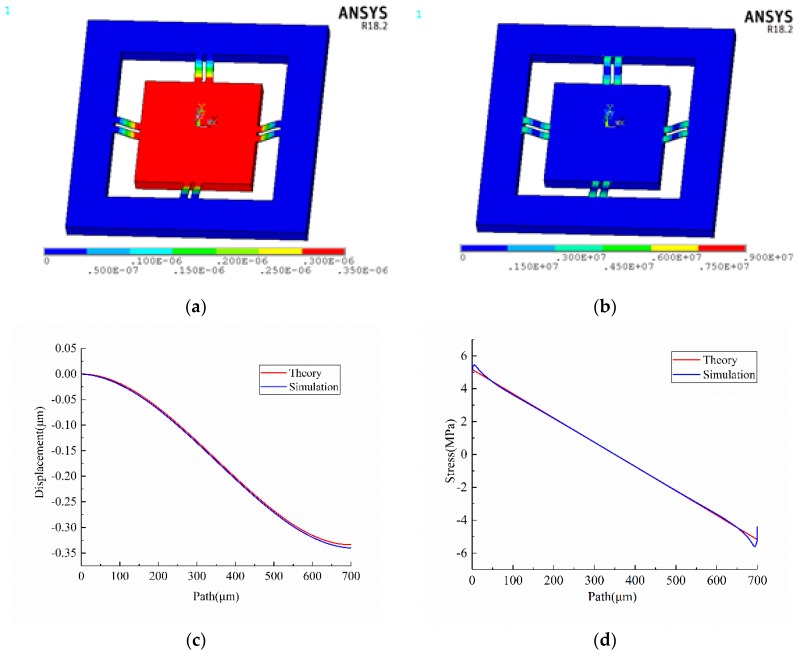
Results when 10 g external acceleration in Z direction (**a**) displacement vector sum (**b**) von Mises stress (**c**) displacement of path P1 (**d**) longitudinal stress of path P1.

The contour plots of displacement vector sum and von Mises stress of the accelerometer experiencing 10 g acceleration in the normal direction are exhibited in Figure 3a,b.

**Figure 4:**
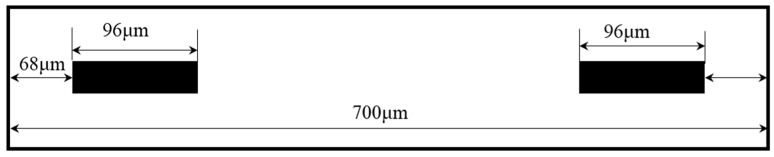
Arrangement of piezo-resistors on beam.

The longitudinal centerline of the upper surface of each beam is defined as a path, such as P1, P2, P2, P3, P4, P5, P6, P7, P8, as shown in Figure 1a. The simulated displacement in Z direction and longitudinal stress of path marked with blue dotted line are plotted in Figure 3c,d. Obviously, they are highly identical with the results calculated by Equations (4) and (5) marked with red solid line. In order to analyze the sensitivity, the following assumptions are made: (1) the length of piezo-resistors is 96 μm; (2) the piezo-resistors are represented by a straight line; (3) the distance between the center of piezo-resistor and nearest end of beams is 116 μm, as shown in Figure 4. Based on previous analysis, the prime sensitivity and cross-axis sensitivities of theory and simulation when an external acceleration in normal (Z) direction are listed in Table 6. The error of prime sensitivity (S_ZZ_) between theory and simulation is 1.59%. The cross-axis sensitivity of the simulation is approximately zero, which is also consistent with the theoretical results.

#### 3.1.2. Acceleration in the X Direction

**Figure 5:**
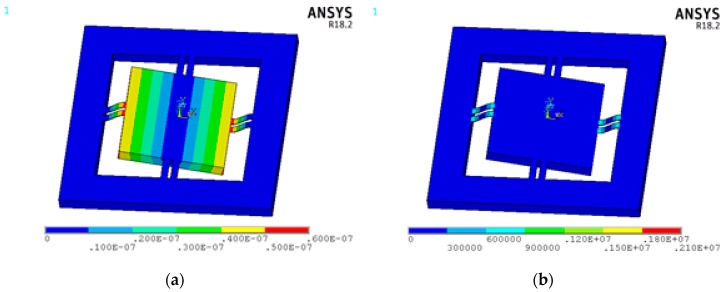

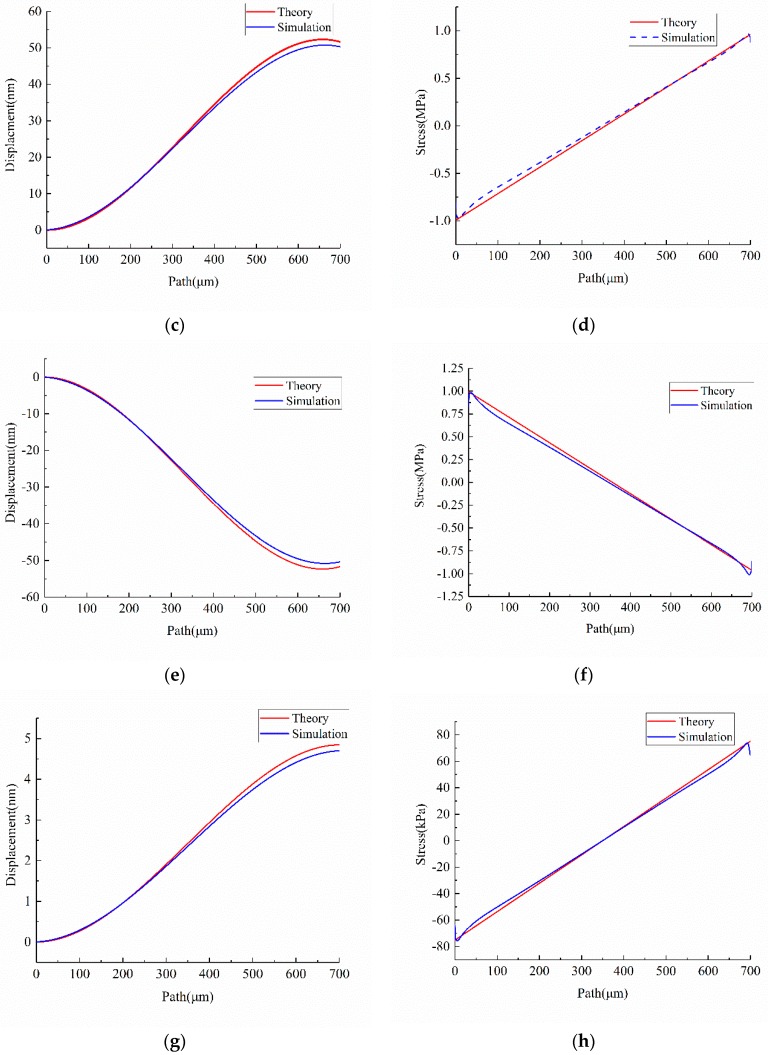

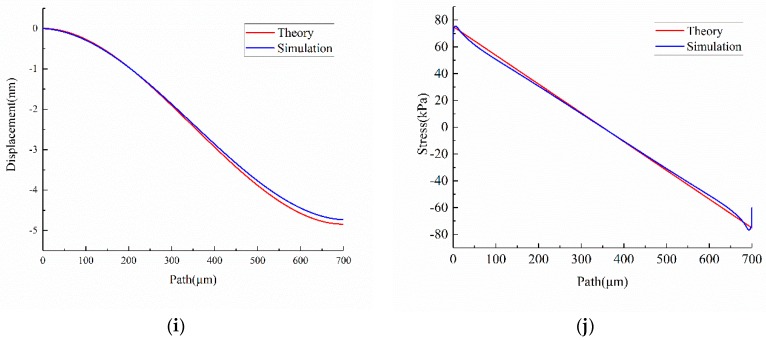
Results under 10 g external acceleration in X direction (**a**) displacement vector sum (**b**) von Mises stress (**c**) displacement in Z direction of path P5 (**d**) longitudinal stress of path P5 (**e**) displacement in Z direction of path P1 (**f**) longitudinal stress of path P1 (**g**) displacement in Z direction of path P4 (**h**) longitudinal stress of path P4 (**i**) displacement in Z direction of path P3 (**j**) longitudinal stress of path P3.

The contour plots of the displacement vector sum and von Mises stress of the accelerometer under 10 g external acceleration in the X direction are shown in Figure 5a,b. The simulated displacement in the Z direction and the longitudinal stress of path P5 and path P1 (X-beams) marked with a blue dotted line are plotted in Figure 5c–f, respectively. The simulated displacement in the Z direction and longitudinal stress of path P4 and path P3 (Y-beams) marked with a blue dotted line are plotted in Figure 5g–j, respectively. The results make it clear that the displacement and stress of X-beams is ten times more than that of Y-beams. In addition, the simulation results are consistent with the theory that is marked with a red solid line.

Then the theoretical and simulation prime sensitivity and cross-axis sensitivity under 10 g external acceleration in the X direction are listed in Table 7. Since the stress of beams under acceleration in the X direction is smaller than that under acceleration in the Z direction, the sensitivity of the X Wheatstone bridge is smaller than that of the Z Wheatstone bridge. The error of the prime sensitivity (S_XX_) between theory and simulation is 6.19%. The cross-axis sensitivities of the simulation are approximately zero, which is also consistent with the theoretical results.

### 3.2. Natural Frequency and Frequency Characteristic

**Figure 6:**
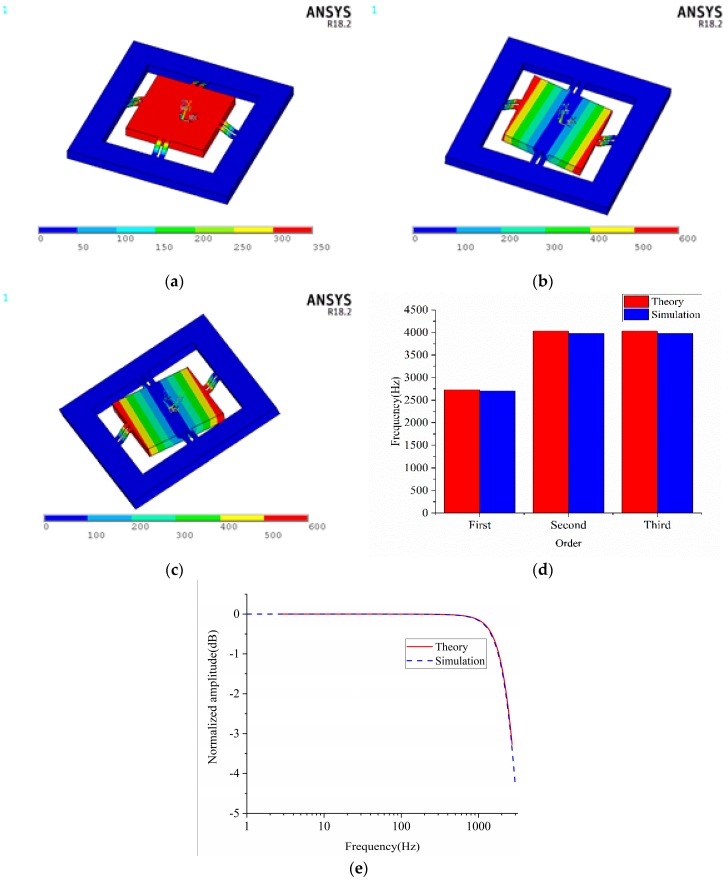
Results of dynamic simulation (**a**) vibration mode of first order natural frequency (**b**) vibration mode of second order natural frequency (**c**) vibration mode of third order frequency (d)natural frequency of calculation and simulation (**e**) amplitude frequency characteristic.

To obtain the natural frequency and vibration modes of the designed accelerometer, modal analyses were conducted. From the simulation results, it is noticed that the proof-mass is vibrating up and down for the first order modal, as shown in Figure 6a, revolving around the Y axis for the second order modal, as shown in Figure 6b and revolving around the X axis for the third order modal, as shown in Figure 6c. The first, second and third order natural frequencies of the simulation are 2702, 3974 and 3976 Hz, respectively. They are pretty close to that calculated by Equations (29) and (33) as shown in Figure 6d. The characteristic amplitude frequency curves are illustrated in Figure 6e. The simulation results are extremely consistent with the theoretical results obtained by Equation (37).

## 4. Results

**Figure 7:**
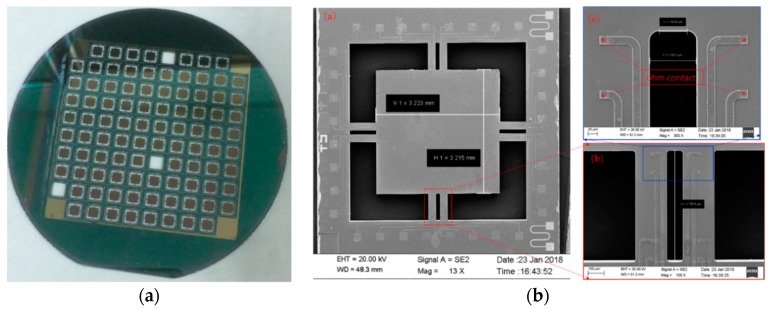
(**a**) Fabricated 4 inches SOI wafer (**b**) scanning electron microscopy (SEM) images of sensor.

The designed accelerometer is fabricated on a silicon-on-insulator (SOI) wafer with an n-type device layer. The resistivity, crystal direction and thickness of the device layer are 3–5 Ω·cm, <100> and 15 μm, respectively. The thickness of the handle layer and oxide layer are 380 μm and 0.5 μm, respectively. The fabrication results are shown in Figure 7a. Figure 7b shows scanning electron microscope (SEM.) images of a fabricated sensor. The measurement results show that the fabricated sensor matches the design value.

**Figure 8:**
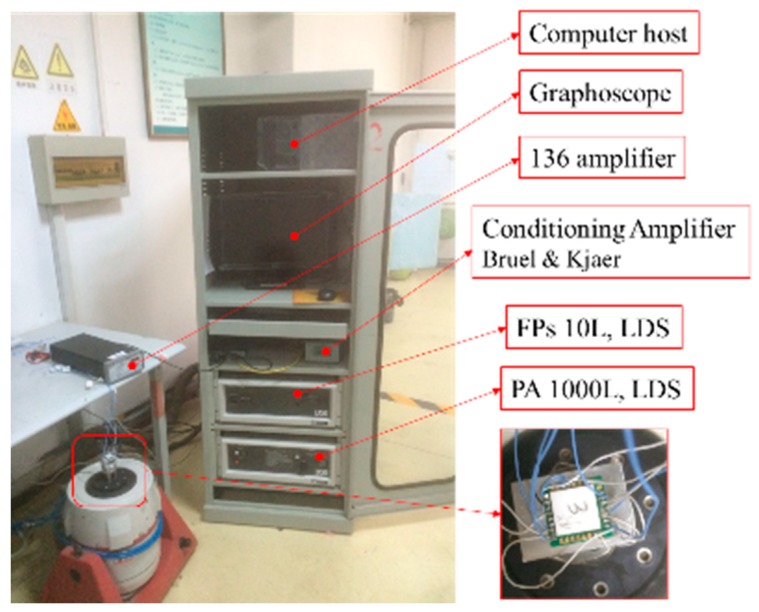
Experimental configuration for testing sensitivity and bandwidth.

The experimental work was conducted on the measurement setup presented in Figure 8. The tested sensor was placed on the vibration platform of a BK3629 vibration and impact sensor calibration system (Denmark). The 136 amplifier can not only magnify the differential signal of the accelerometer by 40 times but also supplies 5 V voltage for the Wheatstone bridge. The magnified output voltage is transmitted to a PC terminal with a customized algorithm for analyzing the performance of the tested chip.

The prime sensitivities of the X Wheatstone bridge at different frequencies are tested. The input accelerations are set at 0.2 g for 2 Hz, 0.5 g for 10 Hz, 1 g for 20 Hz and 40 Hz, 5 g for 80 Hz, 160 Hz, 315 Hz, 630 Hz, 1250 Hz, 2000 Hz and 4000 Hz. However, when the prime sensitivities of the Z Wheatstone bridge at different frequencies are tested, the input acceleration is set at 5 g for 10 Hz, 20 Hz, 80 Hz, 160 Hz, 500 Hz, 1000 Hz, 1200 Hz, 1600 Hz, 2000 Hz and 4000 Hz.

The prime sensitivities and cross-axis sensitivities at 160 Hz of the fabricated accelerometer are listed in Table 8. Error of prime sensitivities S_XX_ and S_ZZ_ between testing and the theoretical value are 7.52% and 4.26%, respectively. In addition, the cross-sensitivities S_XZ_ and S_YZ_ are bigger than S_ZX_ and S_ZY_. This can be explained by Equations (5), (22) and (23). When an external acceleration in the X direction is applied, the absolute longitudinal stress of X-beams at location *x* is not equal to that at location *l-x*; However, the absolute longitudinal stress of the Y-beams direction at location *x* is equal to that at location *l-x.* Under an external acceleration in the Z direction, the absolute longitudinal stress of beam at location *x* is equal to that at location *l-x.*

**Figure 9:**
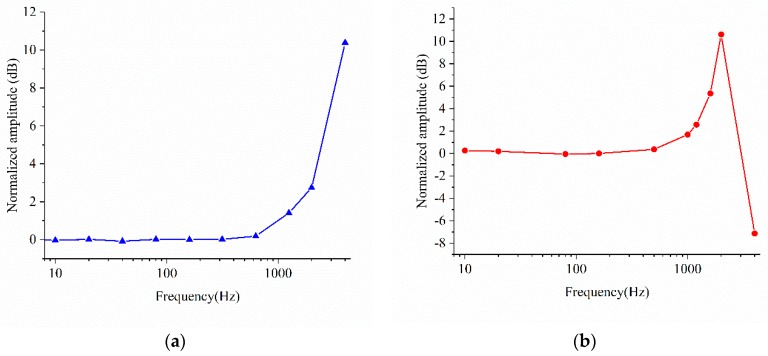
Amplitude frequency characteristic of (**a**) X Wheatstone bridge (**b**) Z Wheatstone bridge.

The amplitude frequency characteristic reference at 160 Hz is obtained by normalizing the prime sensitivities, as shown in Figure 9a,b. The normalized amplitude of the X and Z Wheatstone bridges at 2000 Hz are 2.74 dB and 10.62 dB, respectively. Because the second and third order natural frequency is bigger than that of the first order, the bandwidth of the X Wheatstone bridge is wider than that of the Z Wheatstone bridge. The actual bandwidth is smaller than that of the model. This may be caused by fabrication deviations, particularly because the air damping thickness has a very significant impact on the bandwidth.

## 5. Discussion and Conclusions

The results of the developed mathematical model and FEM show that the displacement and stress of beams when the acceleration is in the Z direction is bigger than that in the X direction. This can account for why S_ZZ_ is bigger than S_XX_ and S_YY_. For the reason that piezo-resistors of the X Wheatstone bridge correspond to that of Y Wheatstone bridge rotated by 90 degrees counterclockwise. $S_{XX}$ is approximately equal to $S_{YY}$. Equation (5) implies that the absolute value of stress at location *x* and *l*-*x* is the same when the is acceleration in the Z direction. As a result, $S_{XZ}$ and $S_{YZ}$ are almost equal to zero. Equations (22) and (23) state clearly that under acceleration in the X direction, the absolute value of the longitudinal stresses of beams along the X direction at location *x* and *l*-*x* are not the same, while, the absolute value of longitudinal stress of beams along the Y direction at location *x* and *l*-*x* is the same. Because no piezo-resistor for detecting acceleration in the Y direction is distributed on the X-beams, in contrast to the two piezo-resistors for detecting acceleration in the Z direction, $S_{ZX}$ is bigger than $S_{YX}$ and $S_{YX}$ is about zero. Based on the developed models, the method of reducing the cross-outputs is appended in Appendix A. 

In this work, the static and dynamic performance of a piezo-resistive eight-beam three-axis accelerometer was studied and analyzed to develop a suitable mathematical model. Further analyses show that the results obtained via the mathematical model closely match the FEM results. According to the mathematical model, an accelerometer is designed and fabricated. Testing results show that the prime sensitivities S_XX_, S_YY_ and S_ZZ_ are 0.209 mV/g, 0.212 mV/g and 1.247 mV/g, respectively. The normalized sensitivity amplitudes at 2000 Hz are 2.74 dB and 10.62 dB, respectively.

## Figures and Tables

**Table 1:** Performance comparison of different accelerometer.

Parameters	Piezoelectric	Piezo-Resistive	Capacitive
DC response	poor	good	good
Impedance	high	low	high
Signal level	high	low	medium
Static Calibration	poor	good	good
sensitivity	high	low	high
Damped design	poor	good	good
Cost	high	low	high
Measurement of long course impact (>10 ms)	bad	good	good
Sensitivity to installation and other stresses	high	low	low
Circuit	simple	simple	complex

**Table 2:** Comparison of predecessors’ work in mathematical models.

Work	Stress	Displacement	Sensitivity	Cross-Axis Sensitivity	Natural Frequency	Amplitude Frequency Characteristics	DoF
Kampen et al. [15]	√ ^a^	√	√	√	√	× ^b^	1
Mukhiya et al. [16]	√	√	√	√	√	×	1
Yu et al. [13]	√	×	×	×	√	×	1
Lin et al. [17]	√	√	√	×	√	×	3
Wang et al. [18]	√	×	√		√	×	1
Meng et al. [19]	√	√	×	×	√	×	3
Wang et al. [20].	√	√	×	×	√	×	1
Hang et al. [21]	×	×	×	×	×	√	3
Liu et al. [8]	√	√	√	×	√	√	1
This work	√	√	√	√	√	√	3

^a^ The symbol √ means that the model of this item is established; ^b^ The symbol × means that the model of this item is not established.

**Table 3:** Stress and change experienced by piezo-resistors for lateral (*X*-axis) acceleration.

Piezo-Resistors	Induced Stress	±ΔR/R
X1	compressive	$-\Delta R_{X1}/X1$
X2	tensile	$+\Delta R_{X2}/X2$
X3	tensile	$+\Delta R_{X3}/X3$
X4	compressive	$-\Delta R_{X4}/X4$
Y1	compressive	$-\Delta R_{Y1}/Y1$
Y2	compressive	$-\Delta R_{Y2}/Y2$
Y3	compressive	$-\Delta R_{Y3}/Y3$
Y4	compressive	$-\Delta R_{Y4}/Y4$
Z1	compressive	$-\Delta R_{Z1}/Z1$
Z2	compressive	$-\Delta R_{Z2}/Z2$
Z3	tensile	$+\Delta R_{Z3}/Z3$
Z4	tensile	$+\Delta R_{Z4}/Z4$

**Table 4:** Stress and change experienced by piezo-resistors for normal (*Z*-axis) acceleration.

Piezo-Resistors	Induced Stress	±ΔR/R
X1	tensile	$+\Delta R_{X1}/X1$
X2	tensile	$+\Delta R_{X2}/X2$
X3	compressive	$-\Delta R_{X3}/X3$
X4	compressive	$+\Delta R_{X4}/X4$
Y1	tensile	$+\Delta R_{Y1}/Y1$
Y2	tensile	$+\Delta R_{Y2}/Y2$
Y3	compressive	$-\Delta R_{Y3}/Y3$
Y4	compressive	$-\Delta R_{Y4}/Y4$
Z1	compressive	$-\Delta R_{Z1}/Z1$
Z2	tensile	$+\Delta R_{Z2}/Z2$
Z3	tensile	$+\Delta R_{Z3}/Z3$
Z4	compressive	$-\Delta R_{Z4}/Z4$

**Table 5:** Structure parameters used for simulation.

Parameters	Description (μm)
beam length/*l*	700
beam width/*w*	200
beam thickness/*t*	15
beam distance/d_b_	100
proof-mass length/*a*	3200
proof-mass width/*a*	3200
proof-mass thickness/*h*	380
frame length/*l_f_*	6600
frame width/*w_f_*	1000
frame thickness/*t_f_*	395

**Table 6:** Comparison of analytical and simulation results for electrical sensitivity.

Parameters	Theory	Simulation
Sensitivity (S_ZZ_) (mV/g)	1.196	1.177
Cross-axis sensitivity (S_XZ_) (mV/g)	0	−6.526 × 10^−4^
Cross-axis sensitivity (S_YZ_) (mV/g)	0	2.287 × 10^−4^

**Table 7:** Comparison of analytical and simulation results for electrical sensitivity.

Parameters	Theory	Simulation
Sensitivity (S_XX_) (mV/g)	0.226	0.212
Cross-axis sensitivity (S_YX_) (mV/g)	0	7.140 × 10^−6^
Cross-axis sensitivity (S_ZX_) (mV/g)	−0.003	−0.001

**Table 8:** Prime and cross-axis sensitivities of fabricated accelerometer.

Sensitivity (mV/g)	Acceleration Direction		
	X	Y	Z
X	0.209	0.003	0.009
Y	0.004	0.212	0.003
Z	0.011	0.014	1.247

**Figure A1:**
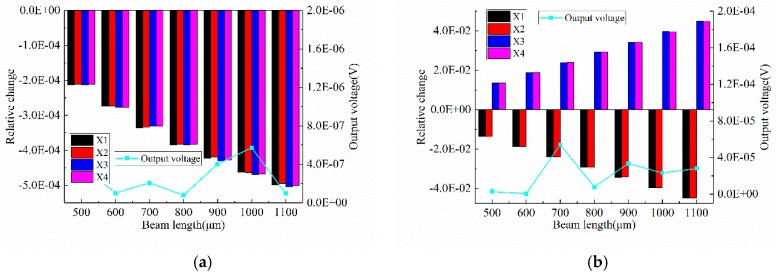
The relative change of piezo-resistors and output voltage of X Wheatstone bridge when acceleration in (**a**) Y direction and (**b**) Z direction.

**Figure A2:**
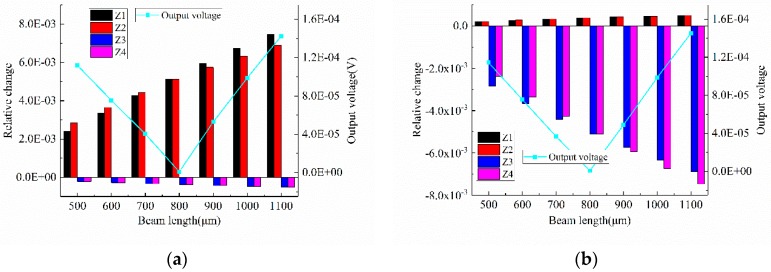
The relative change of piezo-resistors and output voltage of Z Wheatstone bridge when acceleration in (**a**) X direction and (**b**) Y direction.

## Appendix A

In fact, the method of decreasing cross-sensitivity is implicit in Equation (22). Optimization of beam length by enable $\sigma_{2l}\left(x\right)=-\sigma_{2l}\left(l-x\right)$ can effectivity decrease cross-sensitivity. Equation (A1) is obtained by simplified $\sigma_{2l}\left(x\right)=-\sigma_{2l}\left(l-x\right)$:

(A1) $$l\;\frac{6b+\sqrt{36b^{2}+\frac{6h-8t-4t\varepsilon }{t}\left(6b^{2}+6d^{2}\right)}}{\frac{3h}{t}-4-2\varepsilon }$$

In order to verify this, a simulation was conducted. The parameters of the sensor structure for the simulation are listed in Table A1. The relative change of the piezo-resistors and output voltage of the X Wheatstone bridge and Z Wheatstone bridge are shown in Figure A1 and Figure A2, respectively. The left *Y* axis represents the relative change of piezo-resistors and the right *Y* axis represents the absolute output voltage of the Wheatstone bridge. It is obvious that the cross-outputs of $V_{XY}$ and $V_{XZ}$ has little to do with beam length. However, the cross-outputs of $V_{ZX}$ and $V_{ZY}$ are much smaller when the beam length is 800 μm than for any other beam length. These results are consistent with Equations (5), (22) and (23). Unfortunately, the optimal beam length is bigger than the actual beam length we fabricated, but a method of optimizing beam length is obtained, so the beam length will be optimized in the following accelerometer design and processing.

**Table A1:** Structure parameters for simulation.

Parameters	Value (μm)	Parameters	Value (μm)
beam length	500–1100	beam width	200
beam thickness	15	beam distance	300
Proof mass length	3200	Proof mass thickness	380
Piezo-resistors length	96	Piezo-resistors width	8

## References

[1] Biswas S., Gogoi A.K. (2016). Design Issues of Piezoresistive MEMS Accelerometer for an Application Specific Medical Diagnostic System. IETE Tech. Rev..

[2] Sabato A., Niezrecki C., Fortino G. (2016). Wireless MEMS-Based Accelerometer Sensor Boards for Structural Vibration Monitoring: A Review. IEEE Sens. J..

[3] Speller K.E., Yu D. (2004). A low-noise MEMS accelerometer for unattended ground sensor applications. Proc. SPIE.

[4] Chiang C.T. (2018). Design of a CMOS MEMS Accelerometer Used in IoT Devices for Seismic Detection. IEEE J. Emerg. Sel. Top. Circuits Syst..

[5] O’Loughlin C.D., Gaudin C., Morton J.P., White D.J. (2015). MEMS accelerometers for measuring dynamic penetration events in geotechnical centrifuge tests. Int. J. Phys. Model. Geotech..

[6] Santoso D.R., Maryanto S., Nadhir A.J.T. (2015). Application of Single MEMS-accelerometer to Measure 3-axis Vibrations and 2-axis Tilt-Angle Simultaneously. TELKOMNIKA Telecommun. Comput. Electron. Control.

[7] Sankar A.R., Das S., Lahiri S.K. (2009). Cross-axis sensitivity reduction of a silicon MEMS piezoresistive accelerometer. Microsyst. Technol..

[8] Raaja B.P., Daniel R.J., Sumangala K. (2017). A Simple Analytical Model for MEMS Cantilever Beam Piezoelectric Accelerometer and High Sensitivity Design for SHM (structural health monitoring) Applications. Trans. Electr. Electron. Mater..

[9] Dutta S., Saxena P., Panchal A., Pal R., Jain K.K., Bhattacharya D.K. (2018). Effect of vacuum packaging on bandwidth of push–pull type capacitive accelerometer structure. Microsyst. Technol..

[10] Roylance L.M., Angell J.B. (2005). A batch-fabricated silicon accelerometer. IEEE Trans. Electron Devices.

[11] Jung H.I., Kwon D.S., Kim J.J.M., Letters N.S. (2017). Fabrication and characterization of monolithic piezoresistive high-g three-axis accelerometer. Micro Nano Syste. Lett..

[12] Zhao S., Shi Y., Zhao Y., Sun Y., Ren J., Li F. (2018). Design of a Piezoresistive Accelerometer Sensor with Low Transverse Effect. Micronanoelectron. Technol..

[13] Yu X., Zhao L., Jiang Z., Ding J., Peng N., Zhao Y.J.S. (2016). A Novel Piezoresistive Accelerometer with SPBs to Improve the Tradeoff between the Sensitivity and the Resonant Frequency. Sensors.

[14] Liu X.R., Shi K.L., Zou J.L. Multi-Objective Optimization Design for Piezoresistive Accelerometer. Proceedings of the International Conference of the Chinese Society of Micro-Nano Technology.

[15] Van Kampen R.P., Wolffenbuttel R.F. (1998). Modeling the mechanical behavior of bulk-micromachined silicon accelerometers. Sens. Actuators A Phys..

[16] Mukhiya R., Gopal R., Pant B.D., Khanna V.K., Bhattacharyya T.K. (2015). Design, modeling and FEM-based simulations of a 1-DoF MEMS bulk micromachined piezoresistive accelerometer. Microsyst. Technol..

[17] Lin L., Pan F., Xu J., Guo H. Design of a three-axis high-g piezoresistive accelerometer. Proceedings of the IEEE International Conference on Nano/micro Engineered and Molecular Systems.

[18] Wang W., Hao Y. (2003). Design and anslysis of a two-end fixed beam structure of piezoresistive accelerometer. Trans. Microsyst. Technol..

[19] Meng M.Y., Liu J., Shi Y.B. (2007). Structure mechanics analysis on three-axis piezoresistive micro accelerometer. J. Chin. Inert. Technol..

[20] Wang P., Zhao Y., Tian B., Liu Y., Wang Z., Li C., Zhao Y. (2017). A piezoresistive micro-accelerometer with high frequency response and low transverse effect. Meas. Sci. Technol..

[21] Hang B.T., Tan T.D., Duc T.C. (2012). Three-axis piezoresistive accelerometer with adjustable axial resolutions. Vietnam J. Mech..

[22] Doll J.C., Pruitt B.L. (2013). Piezoresistor Design and Applications.

[23] Plaza J.A., Esteve J., Cané C.J.S. (2000). Twin-mass accelerometer optimization to reduce the package stresses. Sens. Actuators A Phys..

[24] Bian Y.M., Zheng F., He H.T. (2008). Research on damping characteristics of MEMS piezoresistive accelerometer. Micronanoelectron. Micronanoelectron. Technol..

[25] Meng L.F., Zheng B. (2005). Sensor Principle and Technology.

